# Safety and Efficacy of Ombitasvir, Paritaprevir With Ritonavir ± Dasabuvir With or Without Ribavirin in Patients With Human Immunodeficiency Virus-1 and Hepatitis C Virus Genotype 1 or Genotype 4 Coinfection: TURQUOISE-I Part 2

**DOI:** 10.1093/ofid/ofx154

**Published:** 2017-07-22

**Authors:** Jürgen K Rockstroh, Chloe Orkin, Rolando M Viani, David Wyles, Anne F Luetkemeyer, Adriano Lazzarin, Ruth Soto-Malave, Mark R Nelson, Sanjay R Bhagani, Hartwig H F Klinker, Giuliano Rizzardini, Pierre-Marie Girard, Cristina Tural, Nancy S Shulman, Niloufar Mobashery, Yiran B Hu, Linda M Fredrick, Tami Pilot-Matias, Roger Trinh, Edward Gane

**Affiliations:** 1 Universitätsklinikum Bonn, Germany; 2 The Royal London Hospital, United Kingdom; 3 AbbVie Inc., North Chicago, Illinois; 4 Denver Health Medical Center, Colorado; 5 Zuckerberg San Francisco General, University of California; 6 Fondazione Centro San Raffaele del Monte Tabor, Milan, Italy; 7 Innovative Care P.S.C., Bayamon, Puerto Rico; 8 Chelsea and Westminster Hospital, London, United Kingdom; 9 Royal Free London Foundation Trust, United Kingdom; 10 Universitätsklinikum Wuerzburg, Germany; 11 ASST Fatebenefratelli Sacco, Milan, Italy; School of Clinical Medicine, Faculty of Health Science, University of the Witwatersrand, Johannesburg, South Africa; 12 Hopital Saint Antoine, Paris, France; 13 Hospital Germans Trias I Pujol, Barcelona, Spain; 14 Liver Unit, Auckland City Hospital, New Zealand

**Keywords:** ART, DAA, HCV, HIV, TURQUOISE

## Abstract

**Background:**

Ombitasvir, paritaprevir with ritonavir, and dasabuvir (OBV/PTV/r ± DSV) ±ribavirin (RBV) are approved to treat hepatitis C virus (HCV) genotype 1 and 4 infection. Here, we investigate the safety and efficacy of OBV/PTV/r + DSV ±RBV for HCV genotype 1, and OBV/PTV/r + RBV for HCV genotype 4, in human immunodeficiency virus (HIV)-1 coinfected patients with or without compensated cirrhosis.

**Methods:**

TURQUOISE-I, Part 2 is a phase 3 multicenter study. Patients with or without cirrhosis were HCV treatment-naive or -experienced, on an HIV-1 antiretroviral regimen containing atazanavir, raltegravir, dolutegravir, or darunavir (for genotype 4 only), and had plasma HIV-1 ribonucleic acid <40 copies/mL at screening. Patients received OBV/PTV/r ± DSV ±RBV for 12 or 24 weeks.

**Results:**

In total, 228 patients were treated according to guidelines. Sustained virologic response at posttreatment week 12 (SVR12) was achieved by 194 of 200 (97%) and 27 of 28 (96%) patients with HCV genotype 1 and genotype 4 infection, respectively. There were 2 virologic failures: 1 breakthrough and 1 relapse in a cirrhotic and a noncirrhotic patient with genotype 1b and 1a infection, respectively. One reinfection occurred at posttreatment week 12 in a genotype 1a-infected patient. Excluding nonvirologic failures, the SVR12 rates were 98% (genotype 1) and 100% (genotype 4). Adverse events were mostly mild in severity and did not lead to discontinuation. Laboratory abnormalities were rare.

**Conclusions:**

The OBV/PTV/r ±DSV was well tolerated and yielded high SVR12 rates in patients with HCV genotype 1 or genotype 4/HIV-1 coinfection. The OBV/PTV/r ± DSV ±RBV is a potent HCV treatment option for patients with HIV-1 coinfection, regardless of treatment experience.

Worldwide, more than 2.2 million people with hepatitis C virus (HCV) infection are estimated to be coinfected with human immunodeficiency virus (HIV)-1 [1]. Coinfected patients remain at a greater risk for HCV disease progression than patients with HCV monoinfection, with an accelerated progression of liver disease leading to cirrhosis and hepatic decompensation; the risk of progression is even greater in coinfected patients with low CD4 T lymphocyte (CD4) cell counts [2–4]. In patients infected with HIV, coinfection with HCV is a leading cause of morbidity and mortality. Studies suggest that although antiretroviral therapy (ART) can reduce the rate of hepatic decompensation in coinfected patients, this rate is still higher than that observed in HCV-monoinfected patients [5, 6]. Therefore, effective HCV treatment in HIV-1-coinfected patients remains a priority.

Until recently, treatment options for coinfected patients still included pegylated interferon (pegIFN) with ribavirin (RBV), which resulted in lower overall efficacy compared with the same treatment in monoinfected patients. The emergence of direct-acting antiviral (DAA) agents has vastly improved the treatment landscape for HCV; most approved regimens report similarly high efficacy rates, regardless of HIV-1 coinfection status [7–11]. The DAAs ombitasvir ([OBV] a NS5A inhibitor) and paritaprevir ([PTV] an NS3/4A protease inhibitor identified by AbbVie and Enanta and codosed with the pharmacokinetic enhancer ritonavir [r]) are approved for the treatment of genotype 4 infection; OBV/PTV/r with the DAA dasabuvir ([DSV] a NS5B nonnucleoside polymerase inhibitor) with or without RBV is approved for treatment of genotype 1 infection [12, 13].

In the phase 2 trial TURQUOISE-I Part 1a, patients with HIV-1/HCV genotype 1 coinfection with or without cirrhosis achieved rates of sustained virologic response (HCV ribonucleic acid [RNA] < lower limit of quantification [LLOQ]) at 12 weeks posttreatment (SVR12) of 94% and 91% after OBV/PTV/r plus DSV treatment for 12 or 24 weeks, respectively [14]. Based on these results, OBV/PTV/r plus DSV was approved for HCV genotype 1/HIV-1 coinfection and recommended in national and international guidelines [7, 8].

TURQUOISE-I, Part 2 is a phase 3 global trial assessing the efficacy and safety of OBV/PTV/r with or without DSV and with or without RBV in an expanded study population of 233 patients with HIV-1/HCV genotype 1 or genotype 4 coinfection with or without cirrhosis.

## METHODS

### Study Design

**Figure 1.**
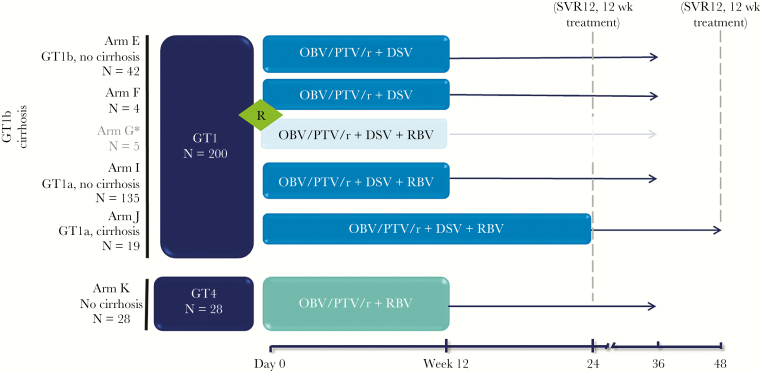
Study design broken down by treatment arm is shown. *Arm G was omitted from this analysis because the recommended regimen for HCV genotype1b patients is ombitasvir, paritaprevir/ritonavir (OBV/PTV/r) plus dasabuvir (DSV) without ribavirin (RBV). GT, genotype; R, randomized treatment arms; SVR12, sustained virologic response at posttreatment week 12.

TURQUOISE-I was a multipart, phase 2 and 3, partially randomized, open-label study. Part 2 was a phase 3 study conducted at 64 sites in the United States, Canada, France, Germany, Italy, New Zealand, Puerto Rico, Russia, Spain, and the United Kingdom. Figure 1 shows the trial profile for Part 2. Studies were designed according to Good Clinical Practice guidelines, Declaration of Helsinki, and applicable regulations, with independent ethics committee or institutional review board approval at all study sites.

### Patients

Patients at least 18 years of age with chronic HCV genotype 1 or genotype 4 infection with HCV RNA >1000 IU/mL at time of screening were enrolled. Eligible patients were HCV/HIV-1 coinfected, with HIV-1 RNA <40 copies/mL (Abbott RealTi*m*e HIV-1 Assay) and on a stable, qualifying HIV-1 ART regimen for at least 8 weeks before screening. Qualified regimens included at least 1 nucleoside/nucleotide reverse-transcriptase inhibitor containing tenofovir disoproxil fumarate, emtricitabine, lamivudine, or abacavir, in combination with 1 or more of the following anchor antiretrovirals: atazanavir coadministered with ritonavir, raltegravir, and/or dolutegravir. Once-daily darunavir coadministered with ritonavir was also allowed for HCV genotype 4-infected patients only, due to a drug-drug interaction (DDI) between DSV and darunavir. However, in a parallel study (TURQUOISE-I, Part 1b), the decrease in the darunavir trough in HCV genotype 1/HIV-1-coinfected patients was not as pronounced as in healthy volunteers [15, 16]. Patients on ritonavir-boosted ART stopped the ritonavir component of their ART regimen during the treatment period and took OBV/PTV/r ± DSV (with or without RBV) concurrently with ART. Details on the dosing of qualified regimens are in the Supplementary Appendix.

Patients with cirrhosis were required to have Child Pugh A (score ≤6) at screening. Cirrhosis status at baseline was defined by liver biopsy (METAVIR score >3 or Ishak score >4), transient elastography (Fibroscan) score ≥12.5 kPa, or serum markers (FibroTest score ≥0.73 and an aspartate aminotransferase-to-platelet ratio index >2). Patients could be (1) HCV treatment-naive or (2) treatment-experienced with pegIFN/RBV or sofosbuvir plus RBV with or without pegIFN.

Exclusion criteria included presence of hepatocellular carcinoma, a positive test result for hepatitis B surface antigen, or infection with any HCV genotype other than genotype 1 or 4. Patients could not have been previously treated with any DAAs other than sofosbuvir. Medications contraindicated for use with OBV/PTV/r ± DSV are listed in Supplementary Table 1. All patients provided signed informed consent before enrollment.

### Procedures and Outcomes

Patients with HCV genotype 1 or 4 and HIV-1 coinfection, with or without cirrhosis, and either HCV treatment-naive or HCV treatment-experienced, received all-oral OBV/PTV/r (25/150/100 mg once-daily) with (genotype 1) or without (genotype 4) DSV (250 mg twice-daily) with or without RBV for 12 or 24 weeks. Shortly after enrollment began, data from the TURQUOISE-III study became available, which demonstrated that 100% of patients with HCV genotype 1b and cirrhosis achieved SVR12 after treatment with OBV/PTV/r + DSV without RBV; these data would later inform the treatment guidelines in this population [17]. Based on these data, enrollment of cirrhotic, genotype 1b patients into the randomized OBV/PTV/r + DSV +RBV treatment arm was stopped, which accounts for the low number of patients (*N* = 5) enrolled in this arm. To align with current treatment guidelines, we present data from those patients with HCV genotype 1b infection who received RBV-free treatment. For patients with HCV genotype 1a or 4 infection, RBV dosing was weight-based at 1000 or 1200 mg divided twice daily, with the exception of patients with creatinine clearance <50 mL/min who received renally adjusted doses of RBV as per label guidelines [18].

The primary endpoint for Part 2 of this study was the proportion of patients achieving SVR12; plasma HCV RNA levels were quantified using the Roche COBAS TaqMan real-time reverse-transcriptase polymerase chain reaction assay, version 2.0. The LLOQ for this assay is 25 IU/mL. Unless otherwise indicated, analyses were conducted in the intention-to-treat population (ITT), which includes all patients who received at least 1 dose of study drug.

The proportion of patients with on-treatment HCV virologic failure, posttreatment relapse, and maintenance of HIV-1 suppression were secondary endpoints. Plasma HIV-1 RNA was quantified using Abbott RealTi*m*e HIV-1 Assay. Failure to maintain HIV-1 suppression was defined as (1) plasma HIV-1 RNA ≥40 copies/mL followed by a repeat HIV-1 RNA of ≥200 copies/mL or (2) 3 consecutive plasma HIV-1 RNA values ≥40 copies/mL.

Analysis of HCV resistance was performed for patients who did not achieve SVR12. Pre-existing amino acid polymorphisms and treatment-emergent amino acid substitutions in HCV NS3, NS5A, and NS5B were identified by population sequencing (detection threshold ~15%). Safety and tolerability were assessed by monitoring of adverse events and conduction of physical examination and clinical laboratory and hematology tests at screening and throughout the study period.

### Statistical Analyses

The primary efficacy endpoint was the proportion of genotype 1-infected patients on a label-recommended regimen achieving SVR12 compared to the SVR12 rate achieved with the standard of care at the time of study design (sofosbuvir plus RBV) for HCV/HIV-1-coinfected patients [10]. Noninferiority to the historical control used a prespecified 10.5% margin and was achieved if the lower confidence bound of the 2-sided 95% confidence interval (LCB) for the percentage of patients achieving SVR12 exceeded 74% (ie, 84%−10.5%). The noninferiority analysis was conducted in the genotype 1 analysis group, defined as all sofosbuvir-naive, genotype 1-infected patients treated per dosing recommendations (all sofosbuvir-experienced patients were excluded from this analysis, as were the 5 cirrhotic, genotype 1b-infected patients who took OBV/PTV/r + DSV + RBV for 12 weeks) [12]. For the primary efficacy endpoint, a sample size of approximately 180 genotype 1-infected patients was calculated assuming an SVR12 rate of 85% to provide 95% power to demonstrate noninferiority compared with the historical control rate with a 2-sided 95% LCB greater than 74% using the normal approximation to the binomial proportion in a 1-sample test. For all efficacy analyses, the confidence intervals (CIs) were 2-sided with a significance level of 0.05 using the Wilson score method for the binomial proportion. The primary analysis was conducted on the ITT population. A sensitivity analysis was performed on the modified ITT-genotype-virologic failure (mITT-GT-VF) populations. This population excluded (1) patients who did not have HCV genotype 1 or genotype 4 infection to prevent any bias occurring from the enrolled population deviating from the population intended to be studied as well as (2) patients who did not achieve SVR12 due to nonvirologic reasons to exclude those failures unrelated to the efficacy of the study drug. Monitoring of adverse events and laboratory abnormalities was performed on all patients who received at least 1 dose of study drug. All statistical analyses were performed using SAS software, version 9.3 (SAS Institute).

## RESULTS

Patients were recruited between July 7, 2015 and November 2, 2015. A total of 332 patients were screened; the primary reason for screen failure was unmet eligibility criteria. Of the 233 patients enrolled and treated, 5 genotype1b-infected, cirrhotic patients who received OBV/PTV/r + DSV + RBV for 12 weeks were excluded from the analyses to align with current label recommendations and European guidelines [7, 12]; 4 of these patients went on to achieve SVR12. Of the remaining 228 patients, 200 had genotype 1 infection and 28 had genotype 4 infection (Figure 1). Patients with genotype 1 infection were mostly genotype 1a (153 of 200; 77%), HCV treatment naive (134 of 200; 67%), and noncirrhotic (177 of 200; 89%). The median baseline CD4^+^ T-cell count was 612 cells/mm^3^ in patients with genotype 1 infection, and 731 cells/mm^3^ in patients with genotype 4 infection. Of the HIV-1 anchor antiretrovirals allowed in this study, raltegravir was the most common (50%) in patients with genotype 1 infection, and dolutegravir was most common (39%) in patients with genotype 4 infection. Complete demographics are shown in Table 1.

**Table 1.** Baseline Demographics

Characteristic^a^	Genotype 1 N = 200	Genotype 4 N = 28
Male, n (%)	156 (78)	26 (93)
White race, n (%)^b^	173 (87)	25 (89)
Black race, n (%)^b^	19 (10)	3 (11)
Hispanic or Latino ethnicity, n (%)^b^	26 (13)	1 (4)
Age, median (range), years	50 (26–69)	47 (30–63)
BMI, median (range), kg/m^2^	25 (17–41)	24 (15–38)
HCV genotype 1a, n (%)^c^	153 (77)	–
IL28B non-CC genotype	133 (67)	21 (75)
Prior HCV Treatment History		
Treatment naive	134 (67)	17 (61)
PegIFN/RBV	62 (31)	11 (39)
SOF-RBV	3 (2)	0
HCV RNA, median (range), log_10_ IU/mL	6.5 (1.8–7.6)	6.0 (4.7–7.0)
CD4^+^ cell count, median (range), /µL	612 (133–2351)	731 (262–1533)
HIV-1 RNA <40 copies/mL, n (%)	193 (97)	27 (96)
Baseline Fibrosis Stage^d^		
F0-1	139 (70)	21 (75)
F2	20 (10)	2 (7)
F3	17 (9)	5 (18)
F4	23 (12)	0
ART Regimen		
Darunavir/r	–	8 (29)
Atazanavir/r	44 (22)	2 (7)
Raltegravir	100 (50)	7 (25)
Dolutegravir	56 (28)	11 (39)
History of injection drug use	105 (54)	4 (15)

Abbreviations: ART, antiretroviral therapy; BMI, body mass index; HCV, hepatitis C virus; HIV, human immunodeficiency virus; PegIFN, pegylated interferon; r, ritonavir; RBV, ribavirin; RNA, ribonucleic acid; SOF, sofosbuvir.

^a^Percentage totals exceeding 100% due to rounding.

^b^Race and ethnicity were self-reported.

^c^Genotypes were determined by the Versant HCV Genotype Inno-LiPA Assay, version 2.0, or Sanger sequencing assay.

^d^One genotype 1-infected patient missing data.

**Figure 2.**
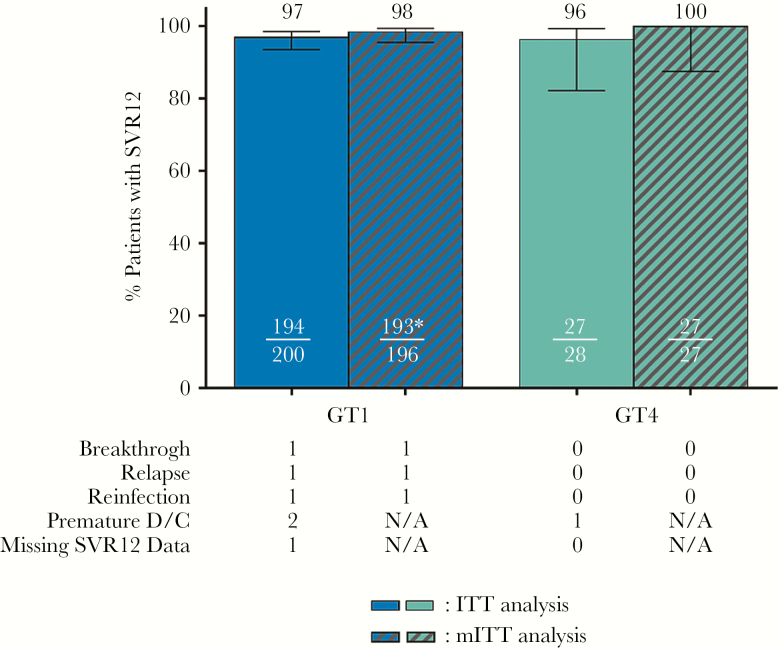
The number and percentage of patients with genotype 1 ([GT1] blue) and GT4 (teal) infection who achieved sustained virologic response at posttreatment week 12 (SVR12) are shown in the intention-to-treat population (ITT) (solid) and modified ITT ([mITT] cross-hatched) populations. *Patient with reinfection is included in the mITT analysis for not achieving SVR12, and not excluded from the denominator as a non-virologic failure. One patient included in the ITT analysis (N = 200) who achieved SVR12 was excluded from the mITT analysis due to having undetermined genotype, bringing N = 99 (minus 3 non-virologic failures, N = 196).

SVR12 was achieved by 194 of 200 (97%; 95% CI, 93.6–98.6) patients with genotype 1 infection, demonstrating noninferiority to the historical control. In the 28 patients with genotype 4 infection, 27 (96%; 95% CI, 82.3–99.4) achieved SVR12 (Figure 2). Two virologic failures occurred. One genotype 1b-infected patient with cirrhosis, a non-CC (TT) *IL28B* genotype, and a prior null response to pegIFN/RBV had on-treatment virologic breakthrough at treatment week 10; this patient was 100% treatment compliant, and drug concentrations from sparse pharmacokinetic samples were consistent with the patient being adherent. One genotype 1a-infected, treatment-naive patient without cirrhosis treated for 12 weeks experienced relapse at post-treatment week 4. In addition, 1 genotype1a-infected patient had a reinfection at post-treatment week 12, as determined by phylogenetic analysis (Supplemental Figure 2). The mITT-GT-VF analysis excluded 1 genotype 4-infected patient who prematurely discontinued treatment and 4 genotype1-infected patients: 2 prematurely discontinued treatment, 1 was lost to follow up, and 1 with undeterminable genotype who enrolled in the genotype 1 treatment group who did go on to achieve SVR12. All 3 patients who prematurely discontinued treatment had HCV RNA below LLOQ at time of discontinuation. Excluding these patients, SVR12 rates were 98% (193 of 196) and 100% (27 of 27) in patients with genotype 1 and genotype 4 infections, respectively.

The SVR12 rates were not notably impacted by HCV subtype, ART regimen, cirrhosis status, or prior treatment experience (Supplemental Figure 3). Although a small number of patients with prior sofosbuvir experience were enrolled, all achieved SVR12.

Population sequencing of the regions encoding HCV NS3, NS5A, and NS5B was conducted on samples taken at baseline and the time of failure from patients who experienced virologic failure. The genotype 1b-infected patient with on-treatment virologic failure had no baseline polymorphisms in NS3 or NS5A, but C316N and S556G were present in NS5B; at the time of failure, Y56H and D168V in NS3, Y93H in NS5A, and C316N and S556G in NS5B were present. There were no baseline polymorphisms or treatment-emergent substitutions found in NS3, NS5A, or NS5B in the genotype1a-infected patient that experienced relapse.

Adverse events were mostly mild in severity (Table 2). There were no discontinuations due to adverse events. Serious adverse events occurred in 9 (4.5%) and 1 (4%) of patients with genotype 1 and 4 infection, respectively (Supplementary Table 2). One genotype 1a-infected patient in the 24-week treatment arm with a previous history of depression experienced serious adverse events of anemia on week 4 and depression on week 17 that were assessed by the treating physician as having a reasonable possibility of being related to RBV and DAAs, respectively. The patient’s RBV dose was adjusted, and the anemia resolved after 5 days. Grade 3 or higher total bilirubin elevations occurred in 27 of 200 (14%) and 2 of 28 (7%) genotype 1 and 4 patients, respectively, and were predominantly indirect; 25 of these patients were on an ART regimen that contained atazanavir. The Grade 3 bilirubin elevations that occurred in the remaining 4 patients were due to RBV-induced hemolysis and, to a lesser degree, paritaprevir inhibition of transporters/enzymes involved with bilirubin metabolism. Of the 182 patients on a RBV-containing regimen, 12% experienced a decline in hemoglobin that resulted in a RBV dose modification.

**Table 2.** Safety and Tolerability

Event, n (%)	Genotype 1 N = 200	Genotype 4 N = 28
Any AE	169 (85)	24 (86)
AEs leading to study drug discontinuation	0	0
Serious AE	9 (5)^a^	1 (4)
AEs in ≥10% of overall patients		
Fatigue	48 (24)	5 (18)
Nausea	42 (21)	5 (18)
Diarrhea	32 (16)	1 (4)
Headache	28 (14)	5 (18)
Insomnia	30 (15)	2 (7)
Pruritus	22 (11)	1 (4)
HGB decrease	19 (10)	4 (14)
HGB decreases leading to RBV modification	18 (9)	3 (11)
AST Grade ≥3 (>5 × ULN)^b^	0	0
ALT Grade 3 (5–10 × ULN)^b^	1 (1)	0
ALT Grade 4 (>10 × ULN)^b^	0	0
Total Bilirubin Grade 3 (3–10 × ULN)	26 (13)	2 (7)
Total Bilirubin Grade 4 (>10 × ULN)	1 (0.5)	0
Patients with Grade 3–4 Bilirubin elevations receiving ATV-containing ART, n/N (%)	23 of 26 (88)	2 of 2 (100)
Hemoglobin Grade 2 (8–10 g/dL)	15 (8)^c^	0
Hemoglobin Grade 3 (<8 g/dL)	0	0

Abbreviations: AE, adverse event; ALT, alanine aminotransferase; AST, aspartate aminotransferase; ATV, atazanavir; DAAs, direct-acting antiviral agents; HGB, hemoglobin; RBV, ribavirin; ULN, upper limit of normal.

^a^One serious AE of depression on day 123 was deemed by the treating physician as having a reasonable possibility of being related to DAAs.

^b^Postnadir.

^c^Fourteen of fifteen patients with HGB reductions were on RBV-containing regimen.

Ten (4%) patients experienced episodes of intermittent HIV viremia (≥40 copies/mL) while taking study drug. The HIV-1 RNA values among these 10 patients never exceeded 200 copies/mL and thus did not meet the criteria for HIV-1 genotypic resistance testing. No patients met prespecified failure to maintain HIV-1 RNA suppression, and no patients required a change to ART while on HCV treatment.

## DISCUSSION

The advent of DAA regimens for HCV treatment has resulted in improved outcomes for patients with HCV/HIV-1 coinfection; many approved therapies recommend the same regimen for coinfected patients as for HCV-monoinfected patients, and the majority of restrictions are regarding baseline HCV RNA and possible DDIs with specific ART [7, 8]. In TURQUOISE-I Part 2, we investigated OBV/PTV/r with or without DSV and with or without RBV in a large cohort of patients with HIV-1 coinfection on atazanavir coadministered with ritonavir, raltegravir, dolutegravir, or for genotype 4-infected patients only, darunavir coadministered with ritonavir. In addition to expanding the patient population to include HCV genotype 4-coinfected patients, Part 2 of the study also expanded the allowed ART regimens to include darunavir for patients with genotype 4 infection and dolutegravir for all patients, in addition to the anchor agents atazanavir and raltegravir that were allowed in Part I of the study [14]. Treatment was based on the label recommendations for patients with HCV genotype 1a, 1b or 4 infection, regardless of HIV-1 status.

Treatment with OBV/PTV/r + DSV ± RBV for genotype 1 and OBV/PTV/r + RBV for genotype 4-coinfected patients yielded SVR12 rates of 97% and 96%, respectively. There were no virologic failures in patients with genotype 4 HCV/HIV coinfection. In the genotype 1a-infected patient with relapse, there were no baseline polymorphisms or treatment-emergent substitutions found in NS3, NS5A, or NS5B. The genotype 1b-infected patient with on-treatment virologic failure had C316N and S556G in NS5B both at baseline and at the time of failure; no baseline polymorphisms were present in either NS3 or NS5A, but Y56H and D168 in NS3 and Y93H in NS5A were present at time of failure. In the patient who relapsed, the lack of baseline polymorphisms or treatment-emergent substitutions is interesting; a pooled resistance analysis of 74 patients with virologic failure in phase 2 and 3 clinical trials investigating OBV/PTV/r +DSV ±RBV revealed that although the majority of patients had treatment-emergent substitutions in at least 1 target, 15% had no treatment-emergent substitutions in any target [19].

The direct-acting antivirals were well tolerated, with no discontinuations due to adverse events. Grade 3 bilirubin elevations were primarily indirect and occurred mostly in patients on either or both an atazanavir-containing ART and RBV-containing regimen; the elevations did not lead to discontinuation nor were they associated with worsening hepatic function. All patients maintained suppression of HIV RNA during the treatment period.

Since the time of study design, several regimens have been approved and many have yielded high SVR rates in patients with HCV/HIV-1 coinfection. Similar to TURQUOISE-I Part 2, the ION-4 study was conducted in patients with HCV genotype 1 or 4 and HIV-1 coinfection, and it included cirrhotic patients and those with prior HCV treatment experience. A 12-week treatment duration with ledipasvir/sofosbuvir resulted in SVR12 rates of 96% in 327 genotype 1-infected patients and 100% in 8 genotype 4-infected patients, with 2 virologic breakthroughs and 10 relapses. All 10 patients who relapsed were black, and black race was the only significant predictor of relapse, occurring in 10 of 115 (9%) black patients [20]. Although cross-study comparisons should be interpreted with caution, it is encouraging that of the 22 black patients enrolled in TURQUOISE-I Part 2, 1 patient experienced relapse, and this was the only relapse in the study; although conclusions cannot be made with statistical accuracy, this observation is confirmed by the results of TURQUOISE-I, Part 1b, wherein 41% (9 of 22) of patients were black, and all achieved SVR12 [16]. Together, these results support previous results indicating that black race is not a predictor of relapse with the OBV/PTV/r plus DSV regimen [21]. The recent C-EDGE CO-INFECTION study conducted in treatment-naive patients with HCV genotype 1, 4, or 6 and HIV coinfection demonstrated that 96% (210 of 218) of patients achieved SVR12 after 12 weeks of treatment with elbasvir/grazoprevir, with 5 patients (2%) experiencing relapse [22]. Of interest is the observational study by Milazzo et al [23], which reported real-life treatment outcomes of 5 different DAA regimens, including OBV/PTV/r plus DSV, in HCV-infected patients with or without HIV coinfection; RBV was used in 38% of patients regardless of regimen and in accordance with EASL guidelines. Safety was consistent across all regimens, and efficacy comparable with that observed in clinical trials. This is particularly relevant because although several DAA regimens have been approved since the time of this study’s design that offer shorter treatment durations or RBV-free treatment options, the availability of these regimens varies widely, particularly in areas with limited resources.

## CONCLUSIONS

Limitations of this study include the small number of patients enrolled in certain subgroups, specifically those with cirrhosis, black race, and prior sofosbuvir experience, and that no genotype 4-infected patients with cirrhosis enrolled. One additional limitation was that nonnucleoside reverse-transcriptase inhibitors were not allowed in the ART regimens.

Human immunodeficiency virus-1 patients on certain stable ART regimens coinfected with HCV genotype 1 or 4 can be successfully treated with OBV/PTV/r with or without DSV, respectively. Treatment with OBV/PTV/r with or without DSV, either with or without RBV, demonstrated favorable efficacy and tolerability in important subpopulations, including those with prior treatment experience and cirrhosis.

## Supplementary Data

Supplementary materials are available at *Open Forum Infectious Diseases* online. Consisting of data provided by the authors to benefit the reader, the posted materials are not copyedited and are the sole responsibility of the authors, so questions or comments should be addressed to the corresponding author.

## Supplementary Material

ofx154_suppl_Supplementary_Material
