# Supplementary material for: Safety and Efficacy of Ombitasvir, Paritaprevir With Ritonavir ± Dasabuvir With or Without Ribavirin in Patients With Human Immunodeficiency Virus-1 and Hepatitis C Virus Genotype 1 or Genotype 4 Coinfection: TURQUOISE-I Part 2
Source: Open Forum Infect Dis. 2017 Jul 22;4(3):ofx154. doi: 10.1093/ofid/ofx154 (PMC5597897; doi:10.1093/ofid/ofx154)
Supplement: ofx154_suppl_Supplementary_Material [file ofx154_suppl_supplementary_material.pdf]

## ONLINE SUPPLEMENT

# Safety & Efficacy of Ombitasvir, Paritaprevir/r ± Dasabuvir With or Without RBV in Patients with HIV-1 and HCV Genotype 1 or Genotype 4 Co-infection: TURQUOISE-I Part 2

Jürgen K Rockstroh, Chloe Orkin, Rolando M. Viani, David Wyles, Anne F. Luetkemeyer, Adriano Lazzarin, Ruth Soto-Malave, Mark R Nelson, Sanjay R Bhagani, Hartwig HF Klinker, Giuliano Rizzardini, Pierre-Marie Girard, Cristina Tural, Nancy S. Shulman, Niloufar Mobashery, Yiran B. Hu, Linda M. Fredrick, Tami Pilot-Matias, Roger Trinh, Edward Gane

**Supplemental Figure 1. Patient Disposition**

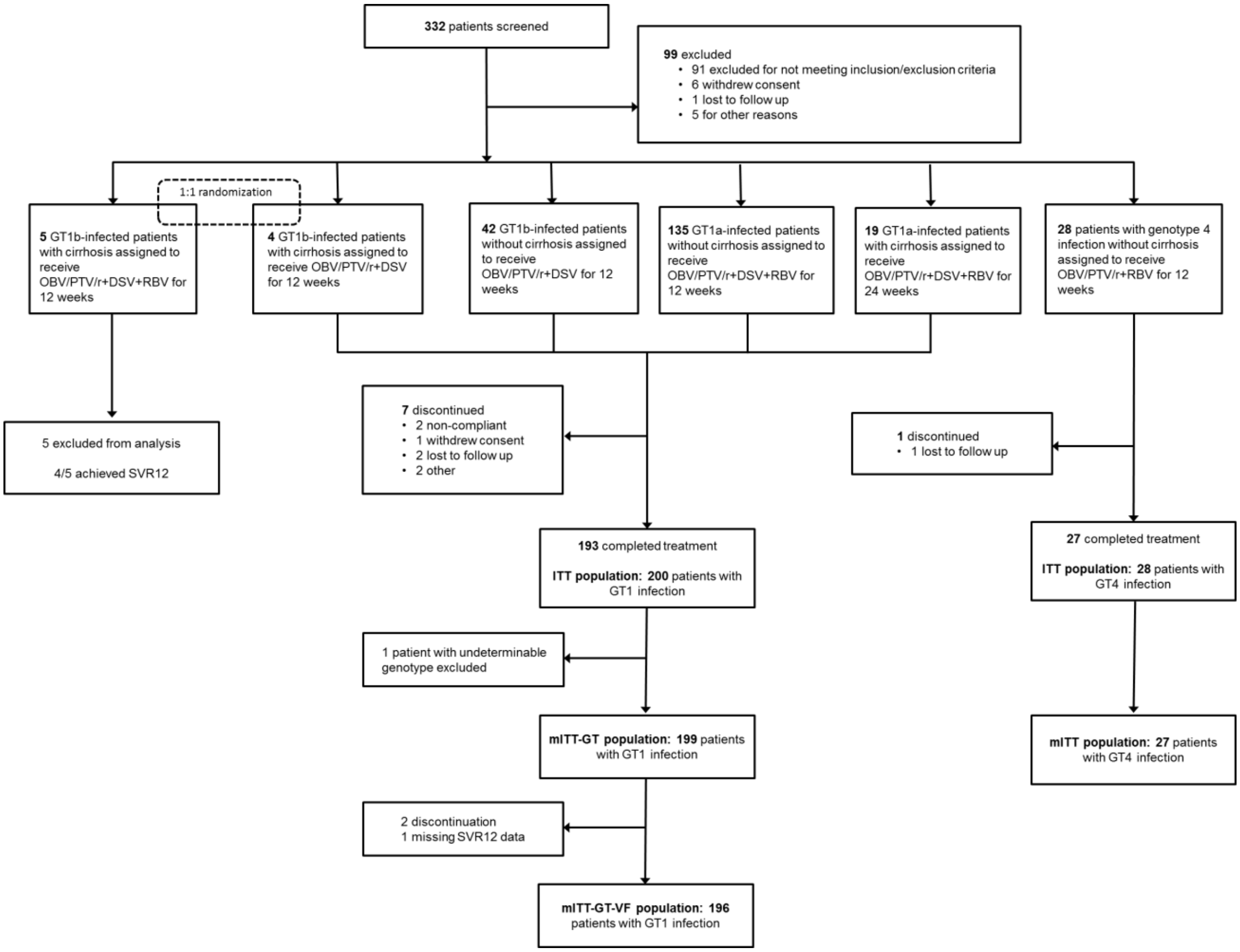


Patient disposition from screening to completion of study period. The ITT population, which includes all patients who received at least one dose of study drug, and modified ITT populations excluding patients with ineligible genotype (mITT-GT) and non-virologic failure (mITT-GT-VF), are shown.

GT, genotype; OBV/PTV/r, ombitasvir, paritaprevir/ritonavir; DSV, dasabuvir; RBV, ribavirin; ITT, intent-to-treat; mITT, modified intent-to-treat; VF, virologic failure.

**Supplemental Table 1. Medications Contraindicated for Use with OBV/PTV/r ± DSV**

| | | |
|---|---|---|
| Alfuzosin<br>Astemizole<br>Carbamazepine<br>Dihydroergotamine<br>**Efavirenz***<br>Ergotamine<br>Ergonovine<br>Fusidic Acid<br>Estrogen-containing Medications for Systemic Use**<br>Gemfibrozil | Lovastatin<br>Methylergonovine<br>Methylergometrine<br>Midazolam (oral)<br>Phenobarbital<br>Phenytoin<br>Pimozide | Rifampin<br>Salmetrol<br>Sildenafil***<br>Simvastatin<br>St. John's<br>Wort<br>Terfenadine<br>Triazolam |

Not all medications contraindicated with HIV-1 antiretroviral agents (ARVs) and ribavirin are listed above. Refer to the most current package inserts or product labeling for a complete list of contraindicated medications.

*** Patients receiving Atripla® (TDF/FTC/efavirenz) or an HIV-1 ART regimen containing efavirenz are not eligible for enrollment.**

** Progestin-only hormonal contraceptive agents are allowed for use with the study drug regimen

*** When used for the treatment of pulmonary arterial hypertension.

- Use of strong or moderate inducers of cytochrome P450 3A (CYP3A) or, for genotype 4 patients only, strong inducers or inhibitors of cytochrome P450 2C8, were prohibited.

**Patient Diagnostic Methods for Cirrhosis**

Liver biopsy: 8 patients

Transient elastography: 191 patients

FibroTest + APRI: 33 patients

Missing data: 1 patient

**HIV-1 ART Dosing**

The HIV-1 ART regimen must include two nucleoside/nucleotide reverse transcriptase inhibitors (NRTIs) plus one of the ritonavir-boosted protease inhibitors noted below, or the integrase inhibitor, raltegravir (RAL). Patients on ritonavir-boosted ART stopped the ritonavir component of their ART regimen during the treatment period and took OBV/PTV/r ± DSV (with or without RBV) concurrently with ART as the 100 mg ritonavir component of the OBV/PTV/r fixed-dose combination provided pharmacokinetic boosting for both PTV and the HIV protease inhibitor.

The nucleoside/nucleotide reverse transcriptase inhibitor combinations in the stable, qualifying ART regimen must be either:

● Tenofovir disoproxil fumarate (TDF) PO QD plus emtricitabine (FTC) PO QD (individual ARV components or as the fixed-dose combination TDF/FTC, Truvada®), or

● Tenofovir disoproxil fumarate (TDF) PO QD plus lamivudine (3TC) PO QD or PO BID (individual ARV components or as the fixed-dose combination TDF/3TC).

The ritonavir boosted protease inhibitor in the stable qualifying ART regimen must be either:

● Atazanavir (ATV) PO QD coadministered with ritonavir (RTV) PO QD, or

● Darunavir (DRV) PO QD coadministered with ritonavir (RTV) PO QD.

The integrase inhibitor in the stable qualifying ART regimen must be:

● Raltegravir (RAL) PO BID.

**Supplemental Figure 2. Phylogenetic Analysis of Patients with Virologic Failure or Reinfection**

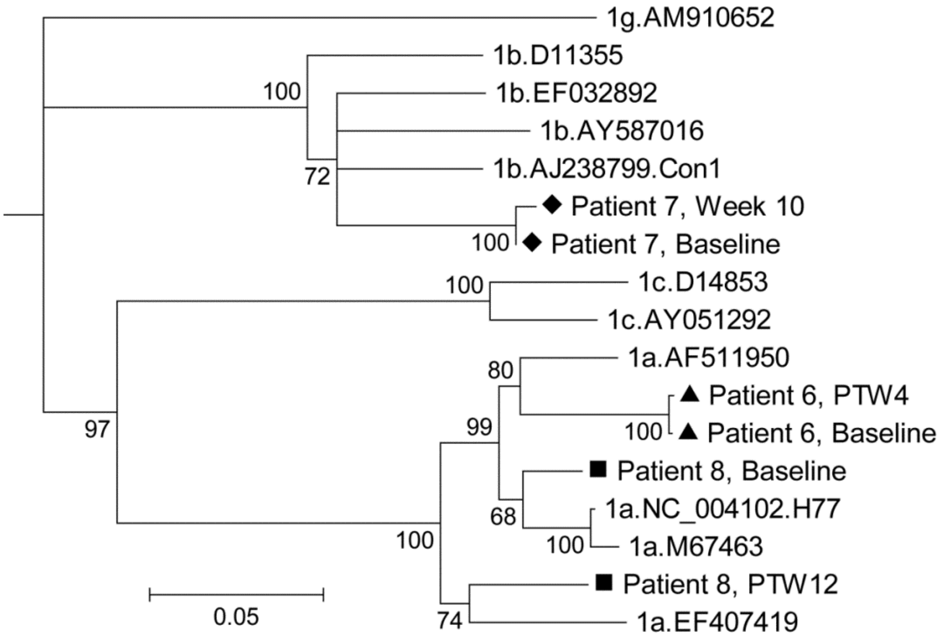


Neighbor-joining phylogenetic tree of NS5A sequences from patients with virologic failure, one each at treatment week 10 (patient 7), post-treatment week 4 (patient 6), and post-treatment week 12 (patient 8). Reliability of the tree topology was examined using 1000 bootstrapping replicates, and bootstrap values ≥50 are listed at appropriate nodes. The genetic distance scale bar indicates the number of nucleotide substitutions per site between sequences. Reference sequences are labeled by the subtype followed by the GenBank accession number. HCV sequences from patients are labeled with the patient number and sample time point. Patient 8 was determined to be re-infected with an isolate of genotype 1a that was distinct from the one present at baseline. Re-infection in the case of the same HCV subtype was defined as a clade switch, as indicated by the lack of clustering between the baseline and post-treatment sequences by phylogenetic analysis.

## Supplemental Figure 3. SVR12 Rates by Subgroup

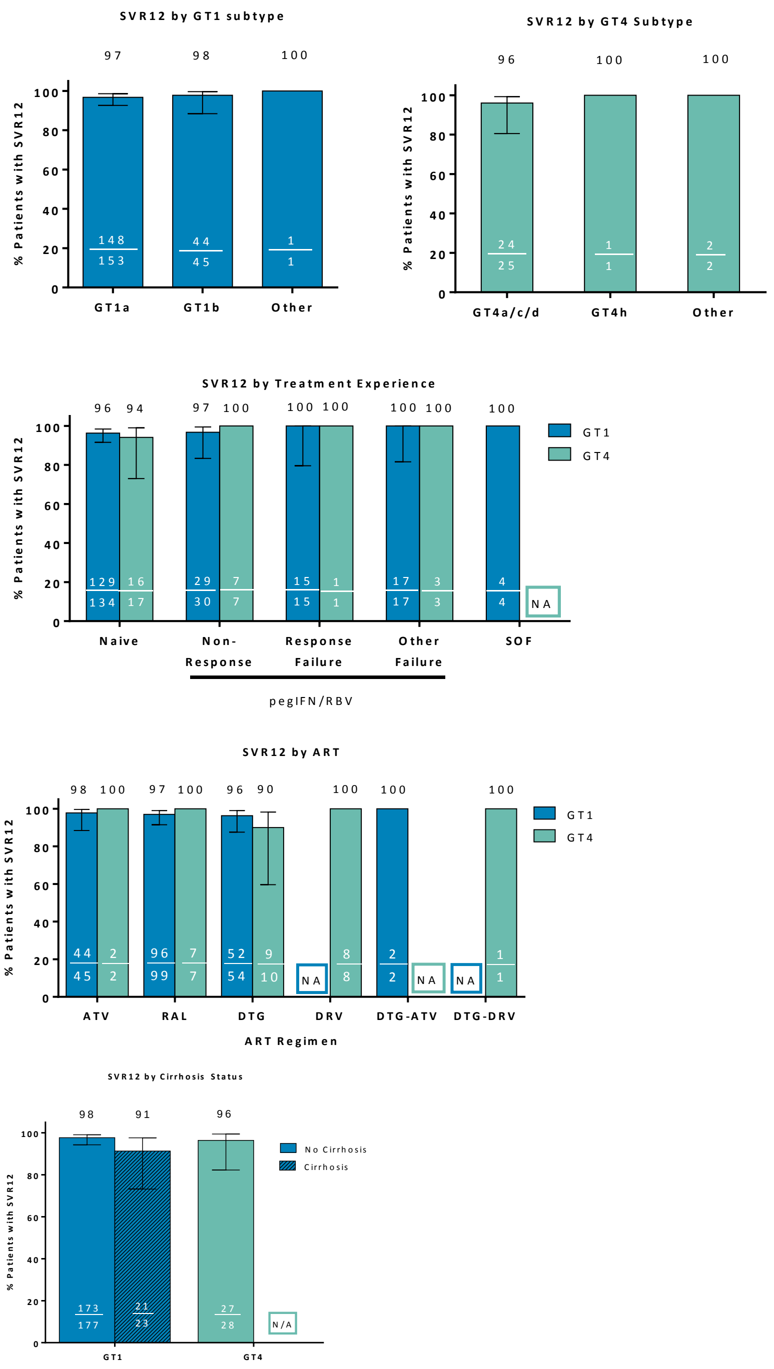


Rates of SVR12 by A) HCV subtype, B) treatment experience C) ART regimen and D) cirrhosis status are shown in the ITT population of patients with genotype 1 or 4 infection.

## Supplemental Table 2. Patients with Serious Adverse Events

| | Adverse Event | Reason Serious | RBV related? | DAA related? |
|---|---|---|---|---|
| 1 | Abdominal pain | Hospitalization | No | No |
| 2 | Rectal perforation | Hospitalization, medical/surgical intervention | No | No |
| 3 | Ureterolithiasis | Hospitalization, medical/surgical intervention | No | No |
| 4 | Overdose | Medical/surgical intervention, life threatening | No | No |
| 5 | Pericarditis | Hospitalization | No | No |
| 6 | Appendicitis | Hospitalization | No | No |
| 7 | Anemia | Hospitalization | Reasonable Possibility | No |
| | Depression | Hospitalization | No | Reasonable Possibility* |
| 8† | Drug dependence | Hospitalization | No | No |
| 9 | Angina unstable | Hospitalization, medical/surgical intervention | NA | No |
| 10 | Hypertensive Crisis | Hospitalization | No | No |

*Deemed by the study sponsor to be unrelated to DAA based on patient history of depression
†Patient with HCV genotype 4 infection
